# Macular hole surgery in times of the COVID-19 pandemic

**DOI:** 10.1038/s41598-025-96528-z

**Published:** 2025-04-13

**Authors:** Nathalie Bleidißel, Julian Klaas, Mathias Maier

**Affiliations:** 1 Department of Ophthalmology, Klinikum rechts der Isar, Technical University Munich (TUM), Ismaningerstr. 22, 81675 Munich, Germany; 2 Department of Ophthalmology, University Hospital Munich (LMU), Munich, Germany

**Keywords:** Full-thickness macular hole, Macular hole surgery, COVID-19 pandemic, Spectral-domain optical coherence tomography, Health-care management, Health services, Eye diseases

## Abstract

To assess whether there were any differences in patient presentation, morphological and functional features as well as surgical treatment for full-thickness macular holes (FTMH) during the lockdown periods amidst the COVID-19 pandemic compared to the previous year. A retrospective analysis was conducted on all patients with FTMH who received treatment at a large tertiary center during the lockdown periods from March 16 to June 16, 2020, and December 9, 2020, to June 6, 2021 (35 eyes, mean age 66 years). Corresponding periods from the previous year were chosen as a control group (41 eyes, mean age 71 years). The patients’ demographics, preoperative and postoperative best-corrected visual acuity (BCVA), symptom duration, time from presentation to surgical treatment, and surgical technique were determined. The minimal and base diameters of the FTMH were assessed using spectral-domain optical coherence tomography. During the lockdown periods in the COVID-19 pandemic, there were no significant differences in the number of patients, duration of symptoms, time from presentation to surgical treatment, surgical technique, macular hole size, base diameter, closure rate or pre- and postoperative BCVA between the two groups. However, there was a significant increase in the number of patients who presented directly at the clinic on an emergency basis without prior contact with a community-based ophthalmologist. This study suggests that the diagnosis and the provision of timely surgical treatment of FTMH were not affected by the COVID-19 pandemic. However, patients more frequently sought immediate emergency care at the hospital. This could be attributed to challenges in scheduling and obtaining appointments at outpatient clinics during the COVID-19 pandemic.

## Introduction

The Coronavirus Disease 2019 (COVID-19) pandemic, caused by Severe Acute Respiratory Syndrome Coronavirus 2 (SARS-CoV-2), has emerged as a significant global health crisis, disrupting healthcare systems worldwide. Lockdowns in 2020 and 2021 led to delays in routine medical care and changes in patient behavior, often due to fear of viral transmission and restricted access to healthcare facilities. Delayed diagnoses and treatments were reported in conditions such as stroke, myocardial infarction, and acute appendicitis, primarily because patients hesitated to seek timely medical attention^1–6^.

In ophthalmology, restrictions on in-person visits and changes in telemedicine utilization can be named^7^. However, surgical management must occur in a face-to-face setting. Therefore, the American Academy of Ophthalmology published guidelines defining emergency surgical procedures during the lockdowns^8^.

In total, these factors may have potentially affected the timely diagnosis and treatment of full-thickness macular holes (FTMH), which is a sight-threatening condition and requires a semi-urgent surgical intervention. Delays in FTMH treatment have been associated with increased hole size and worse functional outcomes^9–14^. A retrospective UK study highlighted the pandemic’s impact on FTMH care, reporting fewer surgeries, increased duration until surgery, larger hole sizes, and worse postoperative outcomes during the COVID-19 period compared to pre-pandemic times. Baseline and postoperative best-corrected visual acuity (BCVA) were reduced, and the failure rate of surgeries increased^15^.

While this is the only study addressing FTMH care during the pandemic, other research has focused on emergency surgeries or anti–vascular endothelial growth factor (anti-VEGF) therapy. A multicenter study on retinal vein occlusion revealed a significant reduction in anti-VEGF injections during the lockdowns, though the postponed care did not significantly affect final BCVA^16^. Another study found a decrease of 75.2% in outpatient visits and 53.6% in anti-VEGF injections compared to the same period in 2019^17^. Delayed anti-VEGF injections led to worse BCVA in neovascular age-related macular degeneration^18^. Studies on retinal detachment showed country-specific differences. UK and US studies reported decreased presentation rates, with more cases presenting as macula-off detachment or with proliferative vitreoretinopathy, leading to worse BCVA^19,20^. In contrast, a German study found no delays in retinal detachment presentation or treatment and no significant difference in preoperative BCVA compared to the previous year^21^.

This study evaluates FTMH patients treated in a large tertiary center in Germany, comparing patient presentation, surgical management, as well as functional and morphological outcomes during the COVID-19 lockdowns to the previous year. It aims to provide insights into the pandemic’s impact on ophthalmology and inform strategies for crisis management, particularly for semi-urgent surgical treatments.

## Methods

### Study design

A retrospective analysis was conducted on consecutive patients with FTMH who presented at the university hospital rechts der Isar of the Technical University Munich, Germany during the lockdown periods due to the COVID-19 pandemic. The two local lockdown periods from March 16 to June 16, 2020, and December 9, 2020, to June 6, 2021, as well as the corresponding periods in 2019 were included. Medical records and spectral-domain optical coherence tomography (SD-OCT, Heidelberg, Spectralis) images were reviewed. This retrospective study was approved by the ethics committee of Technical University of Munich and adhered to the tenets of the Declaration of Helsinki. All participants had given their written informed consent prior to surgery.

### Data collection

For all patients, standard eye examination was performed before surgery. This included BCVA, slit lamp biomicroscopy, intraocular pressure measurements and indirect ophthalmoscopy.

Additional covariates collected were the patient’s age and gender, the duration of symptoms, lens status. The pre- and postoperative best-corrected visual acuity (BCVA), symptom duration, time from presentation to surgical treatment, and surgical technique (with/without phacoemulsification, with/without inverted internal limiting membrane (I-ILM) flap technique) were determined.

SD-OCT images of the macula were conducted. The minimum linear and base FTMH diameter was measured on their narrowest point parallel to the retinal pigment epithelium (RPE) using the manual caliper software tool. Further, FTMH were classified into three subgroups according to the International Vitreomacular Traction Study Group Classification System depending on the minimal linear diameter as follows: small ($\:\le\:\:$250 µm), medium (251–400 μm) and large ($\:>$ 400 μm)^22^.

### Surgical procedure

Standard three-port vitrectomy was performed using a 23-gauge system (D.O.R.C, Düsseldorf, Germany) in all patients. Combined surgery with phacoemulsification and intraocular lens (IOL) implantation was conducted if a significant cataract was present. After core and peripheral pars plana vitrectomy (PPV), the ILM was stained with 0.025% Brilliant Blue G (Brilliant Peel, Fluoron, Germany). Afterwards, either the inverted ILM flap technique or the conventional ILM peeling was performed. At the end of the surgery, a gas tamponade (SF6 or C3F8) was substituted in all cases. All patients were instructed to maintain a face-down position for three days after surgery.

### Statistical analysis

SPSS (version 28.0; SPSS Inc., Chicago, Illinois, USA) was used for the statistical analyses. The decimal visual acuity was converted to the logarithm of the minimum angle of resolution (LogMAR). Continuous variables were reported as the mean ± standard deviation (SD) or median and range, whereas categorical variables were expressed as percentages and absolutes. Two-tailed t-test, Chi-square test or Mann-Whitney U-tests were used for comparison of variables between the two independent groups depending on the variables evaluated. Differences between the two groups were examined at a significance level of *p* < 0.05.

## Results

In total, 35 eyes of 35 patients with FTMH, which received treatment at our clinic during the lockdown periods were included in this retrospective study (lockdown group). In the corresponding periods from the previous year, 41 eyes of 41 patients were included (control group). There was no significant reduction in patients with FTMH presenting and receiving surgical treatment during the lockdown periods (*p* > 0.05).

The analysis revealed no significant differences in patient demographics between the two groups. The mean age of patients in the lockdown group was 66 ± 9 years, while the mean age of patients in the control group was 71 ± 8 years. 54% (*n* = 19) of the patients were female in the lockdown group compared to 59% (*n* = 24) female patients in the control group. This suggests that the patient population remained similar during both periods, indicating consistency in the characteristics of patients with FTMH.

At first presentation, 23 (67,6%) eyes of the lockdown group and 29 (71%) eyes of the control group were phakic (*p* > 0.05). Combined phacoemulsification and intraocular lens implantation with PPV were performed in 17 (48,6%) and 14 (34%) cases, respectively. The surgical technique (with/without phacoemulsification, with/without I-ILM flap technique) did not differ significantly between the two groups (*p* > 0.05). There were no adverse events during surgery in both groups. Postoperative closure rate was 100% in both groups.

**Fig. 1:**
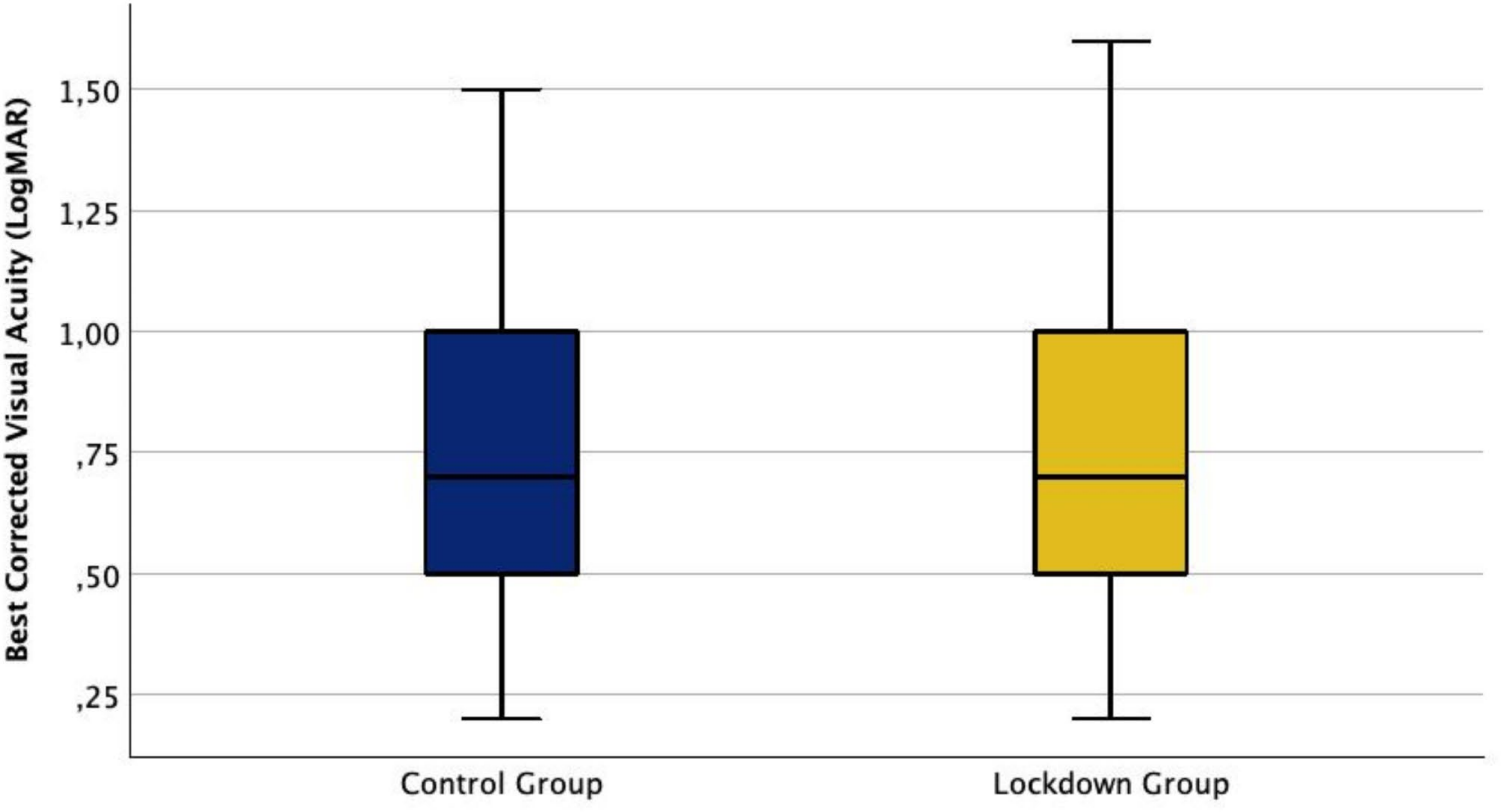
These box plots illustrate the distribution of the preoperative Best Corrected Visual Acuity (BCVA) of the control group and the lockdown group. The median BCVA did not differ between the two groups as it was 0.70 LogMAR in both groups.

The mean preoperative BCVA was 0.76 ± 0.35 LogMAR in eyes treated during the lockdown periods and 0.74 ± 0.30 LogMAR in eyes of the control group. There was no significant difference concerning preoperative BCVA between the two groups (*p* > 0.05, Fig. 1).

**Fig. 2:**
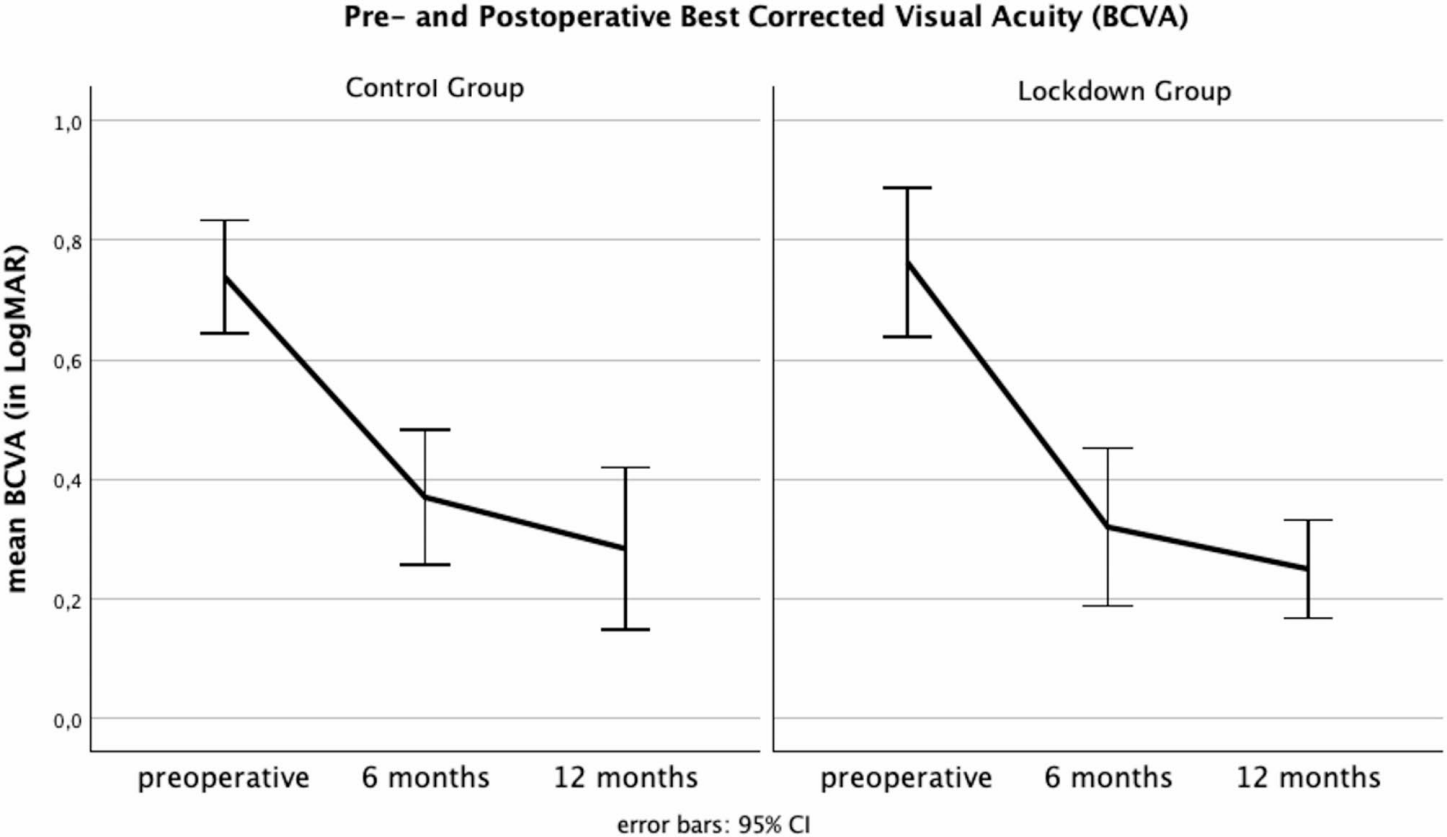
Pre- and postoperative best-corrected visual acuity (BCVA) in LogMAR in the control group (left) and lockdown group (right) at preoperative, six months, and twelve months follow-up. Error bars represent the 95% confidence intervals. Both groups demonstrate significant improvements in BCVA postoperatively, with comparable outcomes at six and twelve months.

We found a statistically significant correlation between the preoperative BCVA and the FTMH minimal diameter as well as the FTMH base diameter. A larger FTMH minimal diameter as well as a larger FTMH base diameter was connected to a worse BCVA (*r* = 0.56, *r* = 0.50, *p* < 0.01). Both groups showed significant postoperative improvements in BCVA, with comparable outcomes at six and twelve months (Fig. 2). At the six-month follow-up, the BCVA was 0.37 ± 0.29 LogMAR in the lockdown group and 0.38 ± 0.24 LogMAR in the control group. At twelve months, it improved further to 0.23 ± 0.16 LogMAR and 0.30 ± 0.26 LogMAR, respectively. These differences between the groups were not statistically significant (*p* > 0.05).

**Fig. 3:**
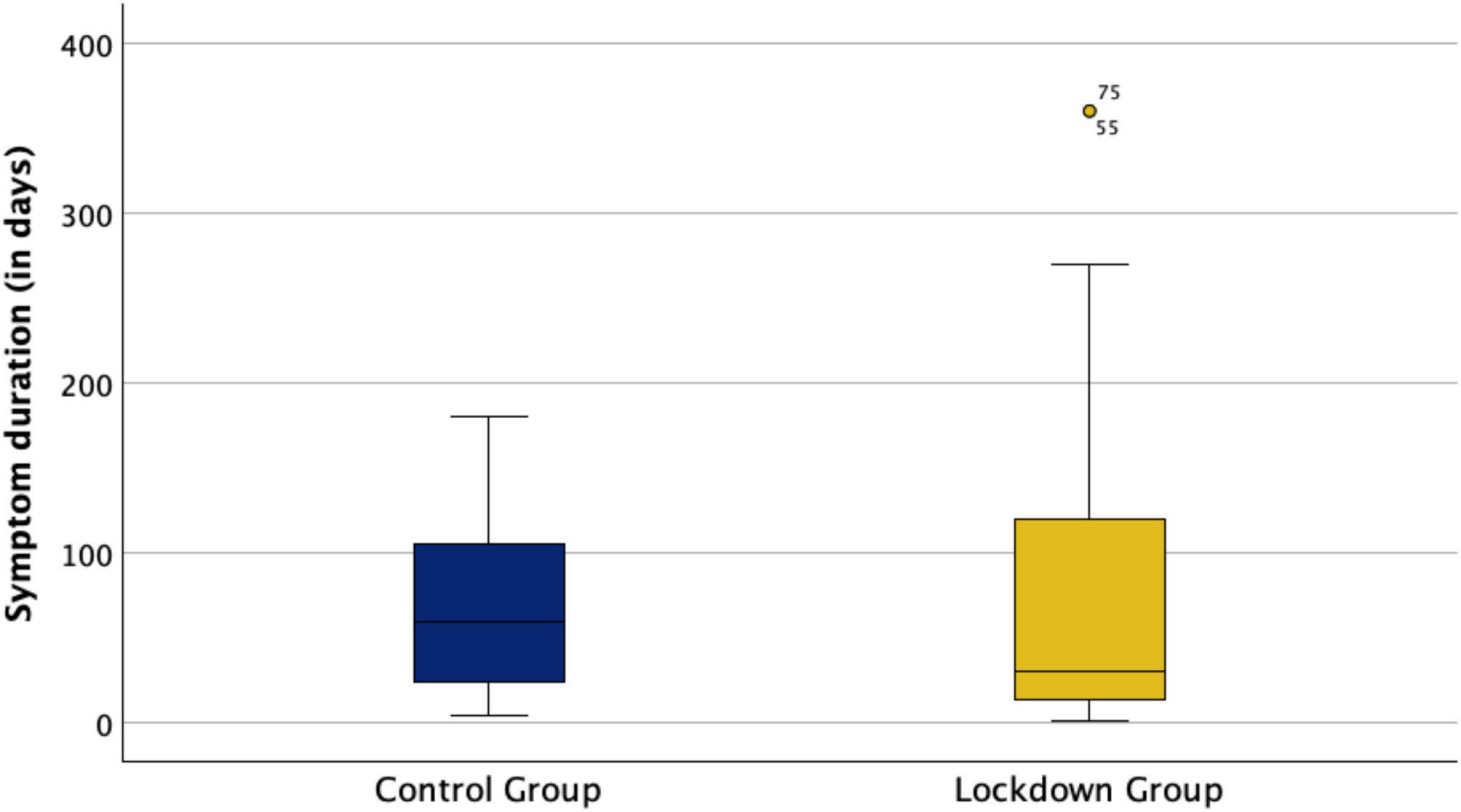
Boxplot showing the distribution of symptom duration (in days) for patients in the control group and in the lockdown group. The median symptom duration did not differ significantly between the two groups (*p* > 0.05). It was 60 days in the control group and 30 days in the lockdown group. The lockdown group exhibits greater variability, with a wider interquartile range and several outliers.

**Fig. 4:**
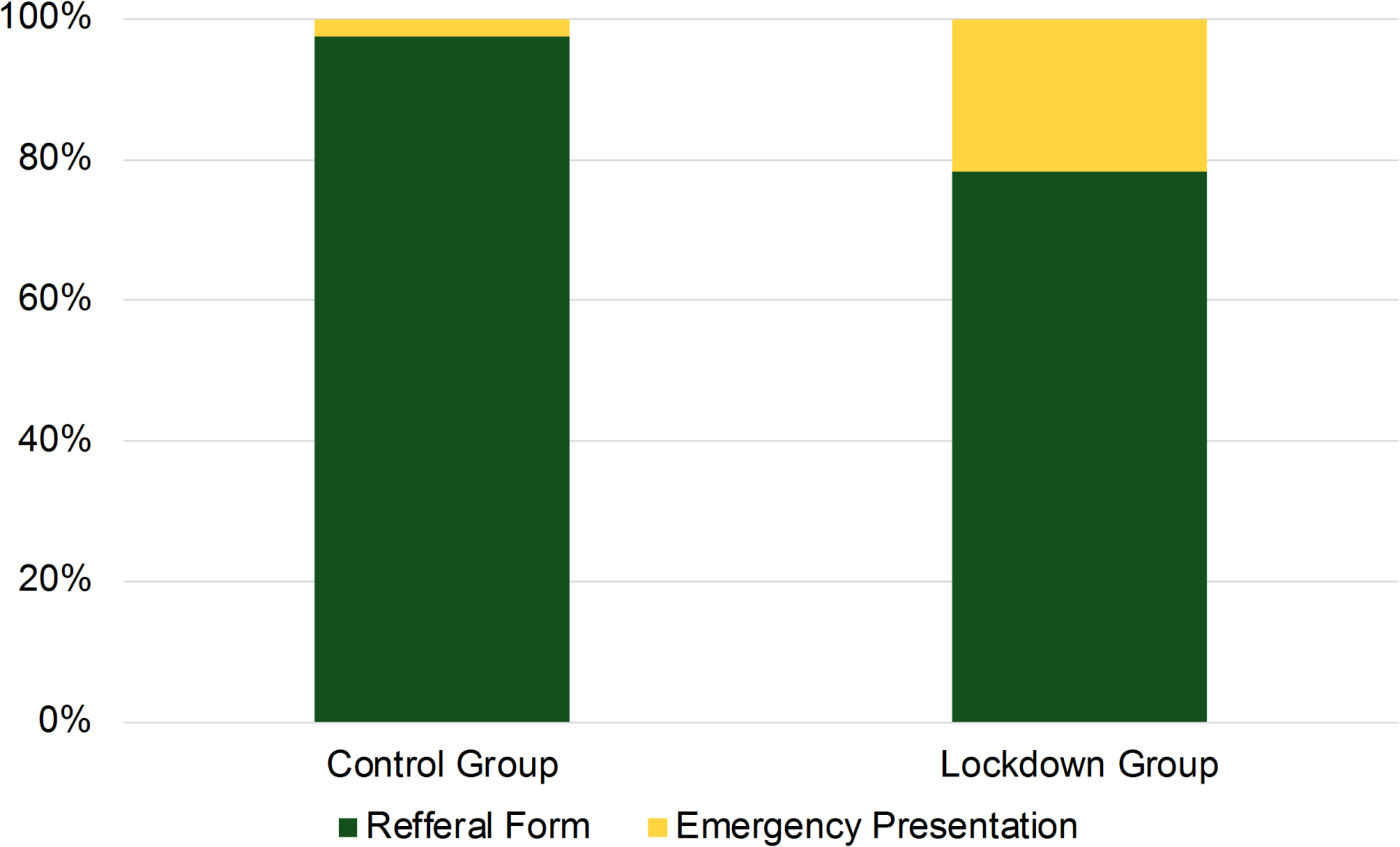
The bar chart compares the proportion of patients presenting themselves on an emergency basis at the clinic and patients referred to the clinic with a referral form an external ophthalmologist between the lockdown group and the control group. Patients of the lockdown group were significantly more likely to present directly at the clinic on an emergency basis (21,7%, *n* = 5) compared to patients of the control group (2,6%, *n* = 1, *p* < 0.01).

The recorded median duration of symptoms did not differ significantly between the two groups. The median duration was 30 days in the lockdown group, whereas it was 60 days in the control group (*p* > 0.05, Fig. 3). This indicates no longer waiting times for an appointment in the clinic during the lockdown periods. A longer symptom duration was connected to a larger mean minimal as well as base FTMH diameter in both groups (*p* > 0.05). However, patients of the lockdown group were significantly more likely to seek help directly at the clinic (21,7%, *n* = 5 vs. 2,6%, *n* = 1), presenting themselves on an emergency basis without a referral, and thus without prior contact with a community-based ophthalmologist (*p* < 0.01, Fig. 4). Interestingly, the time to surgery did no differ significantly between the two groups. The median was 21 days in the lockdown group and 22 days in the control group until surgery (*p* > 0.05).

**Fig. 5:**
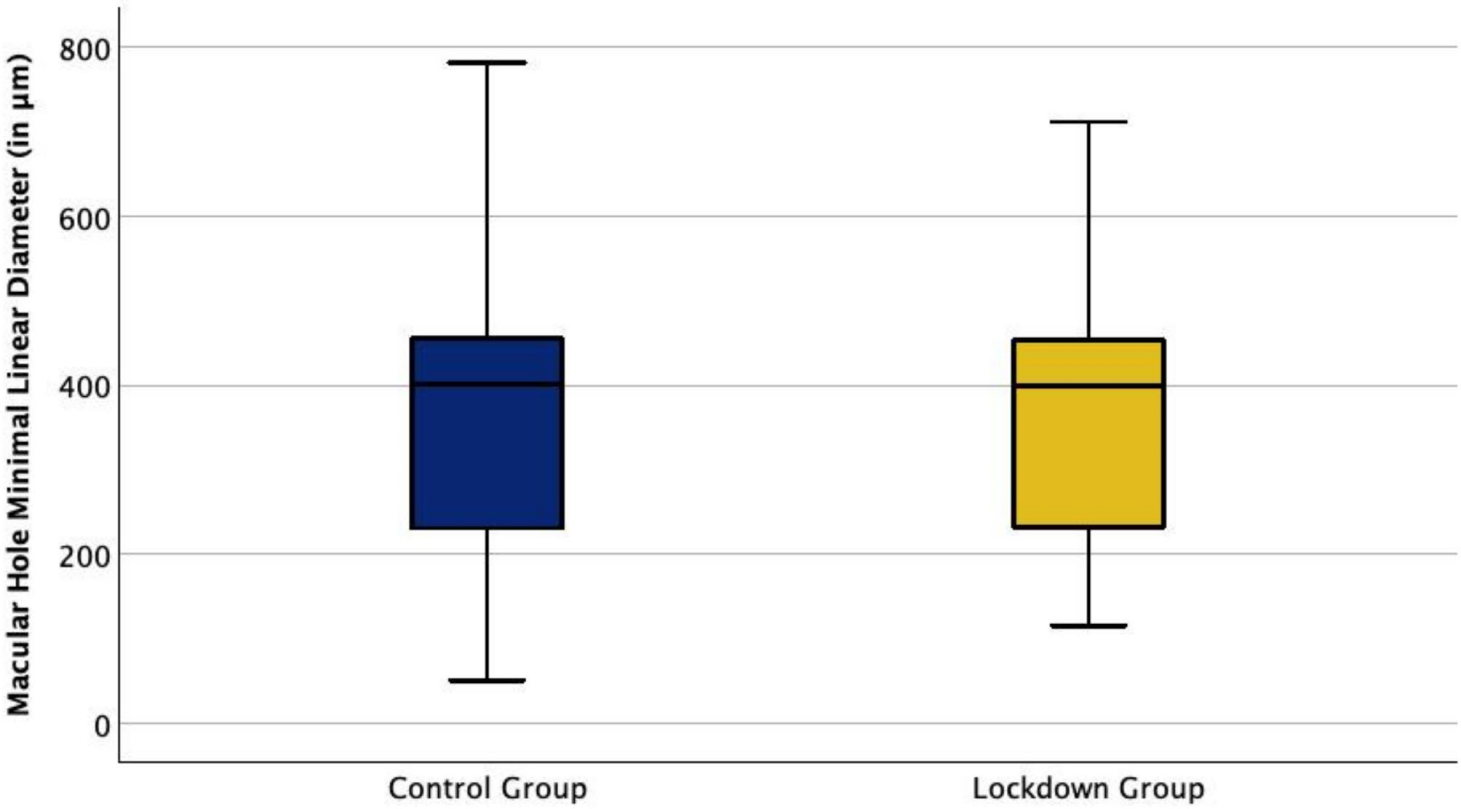
These box plots illustrate the distribution of Macular Hole Minimal Linear Diameter (in µm) of the control group and the lockdown group. The median Macular Hole Minimal Linear Diameter did not differ significantly between the two groups, with 401 μm in the control group and 399 μm in the lockdown group (*p* > 0.05).

The mean minimal FTMH diameter at baseline was 370 μm (± 161 μm, range 116–711 μm) in the lockdown group, whereas it was 386 μm (± 177 μm, range 51–781 μm) in the control group (Fig. 5).

Divided into subgroups, 10 (28,6%) small (< 250 μm), 8 (22,9%) medium (≥ 250 μm) and 17 (48,6%) large FTMH (≥ 400 μm) were treated during the lockdown periods, contrasted against 11 (26,8%) small (< 250 μm), 9 (22%) medium (≥ 250 μm) and 21 (51,2%) large FTMH (≥ 400 μm) in the control group. The subgroup composition did not differ significantly between the two groups (*p* > 0.05). The mean FTMH base diameter was 759 μm (± 355 μm, range 157–1490 μm) in the lockdown group compared to 815 μm (± 354 μm, range 359–1577 μm) in the control group (*p* > 0.05). The mean central retinal height was 393 μm (± 83 μm, range 209–603 μm) in the lockdown group and 415 μm (± 79 μm, range 234–603 μm) in the control group (*p* > 0.05). Concluding, the morphological characteristics did not differ significantly between the two groups. Patients’ demographics and morphological characteristics of the two groups are depicted in Table 1.

**Table 1:** Characteristics of patients of the lockdown group (*n* = 35) and the control group (*n* = 41).

	Lockdown Group	Control Group
Number of patients/eyes	35/35	41/41
Age, years (mean $\:\pm\:$ SD)	66 ($\:\pm\:\:$9.0)	71 ($\:\pm\:\:$8)
Female gender, n (%)	19 (54%)	24 (59%)
Lens status, phakic	23 (67,6%)	29 (71%)
Combined surgery	17 (48.6%)	14 (34%)
I-ILM Flap surgery	19 (54,3%)	27 (65,9%)
Mean duration of symptoms in days (median)	30	60
Mean duration until surgery in days (median)	21	22
Mean FTMH minimal linear diameter in µm ± SD, range	370 (± 161), 116–711	386 (± 177), 51–781
Mean FTMH base diameter in µm ± SD, range	759 (± 355), 157–1490	815 (± 354), 359–1577
Preoperative BCVA (Mean LogMAR ± SD), Snellen	0.76 (± 0.35) 20/125	0.74 (± 0.30) 20/100
Postoperative BCVA after 6 months (Mean LogMAR ± SD), Snellen	0.37 (± 0.29) 20/50	0.38 (± 0.24) 20/50
Postoperative BCVA after 12 months (Mean LogMAR ± SD), Snellen	0.23 (± 0.16) 20/32	0.30 (± 0.26) 20/40

I-ILM = inverted internal limiting membrane; FTMH = full-thickness macular hole;

BCVA = best-corrected visual acuity; LogMAR = logarithm of minimal angle of resolution;

SD = standard deviation.

## Discussion

The emergence of the COVID-19 pandemic has necessitated changes in healthcare delivery to ensure patient and healthcare provider safety while minimizing the spread of the virus. Various restrictions led to cancellations of routine appointments and surgery in an inpatient as well as in an outpatient setting in multiple medical fields^3–6^. To our knowledge, this study is the first to assess the impact of the lockdown periods amidst the COVID-19 pandemic on ophthalmic surgery care addressing a semi-urgent ocular condition in a large tertiary center. Patients with a FTMH require semi-urgent intervention to optimize morphological and functional outcomes. Several studies have demonstrated the negative impact of delayed macular hole surgery on visual and morphological outcomes^8–13^. We selected patients with a FTHM as an index disease since the impact of the COVID-19 pandemic has not yet been examined for semi-urgent ocular conditions.

The findings of this study indicate that there were no significant differences in the number of patients presenting with FTMH during the lockdown periods compared to same periods of the previous year. This suggests that the overall incidence of patients presenting with FTMH remained relatively stable despite the challenging circumstances due to the COVID-19 pandemic. Also, the duration of symptoms, which reflects the time from the onset of symptoms to seeking medical attention, showed no significant differences between the lockdown and control group. This indicates that patients did not delay seeking medical care for their ocular complaints during the lockdown periods. Regarding the time from presentation to surgical treatment, there were no significant differences observed between the two groups. This suggests that despite the challenges posed by the COVID-19 pandemic, patients with FTMH were able to receive timely surgical intervention. Therefore, we state that despite potential negative influences of the lockdown periods on the delivery of eye care services (e.g., providing elective appointments, reduced healthcare staff due to disease), management and treatment for a semi-urgent ocular condition like FTMH has not been severely affected in a large tertiary-center in Germany. Further, our data indicates that the lockdown period did not negatively impact visual improvement after macular hole surgery over a time of twelve months postoperatively. A similar study conducted in the US focusing on retinal detachment, found comparable rates of single surgery anatomical success and final BCVA during the pandemic and pre-pandemic, with no delays in surgical intervention^23^.

However, in this study the rate of emergency presentations without prior consultation with a community-based ophthalmologist in an outpatient setting increased significantly during the lockdown periods compared to the control group. This is an important information which emphasizes the need for large centers for ophthalmology to compensate such a shift during a pandemic. It may be attributed to difficulties in obtaining appointments or scheduling consultations during the lockdown periods in outpatient clinics during the COVID-19 pandemic. Delayed presentation due to fear of infection or difficulties in obtaining appointments were found in comparative research for retinal detachment. Also, a higher prevalence of macular off retinal detachment, more proliferative vitreoretinopathy and worse BCVA were reported during the COVID-19 pandemic^24–27^.

Primary care in Germany is characterized by short waiting times and readily accessible services in large clinics. While barriers to healthcare access can exist in some cases, such as variations based on insurance status, age, or region of residence, this was not observed in our study^28,29^. At our clinic, patients with FTMH are treated as semi-urgent cases and are evaluated on the same day, with surgery scheduled promptly, regardless of insurance status. During the lockdown admits the COVID-19 pandemic, patients had 24/7 access to care at our clinic and were promptly evaluated. Semi-urgent surgeries, including macular hole repair, were also consistently performed without delays during the lockdown periods. Notably, a large study from the UK reported an average symptom duration of approximately three months for macular hole patients, even outside of pandemic times, compared to one to two months in our study^9^. This highlights the structural differences between systems such as the NHS in the UK and Germany, where strong infrastructure and financial investment allow for consistent access and shorter waiting times, even during crises.

In this study we further confirmed prognostic factors concerning morphological features of FTMH and BCVA. A larger FTMH minimal diameter as well as a larger FTMH base diameter were both independently connected to a worse BCVA. Further, our findings suggest that longer symptom duration is associated with larger minimal and base FTMH diameters, which in turn correlate with worse BCVA. The FTMH diameter acts as a mediator variable in this relationship, as it directly impacts functional outcomes. This highlights the importance of a timely diagnosis and intervention for patients with FTHM to minimize the progression of FTMH diameter and its impact on visual function^9–14,31^.

Understanding the challenges in eye care delivery is crucial for optimizing patient care during a pandemic and future healthcare planning. Continued research, implementation of infection control measures and prioritization protocols, as well as the integration of telemedicine into routine eye care practice will be essential in navigating the challenges posed by future pandemics and ensuring the provision of safe and effective eye care services^7,28^. Large centers, such as ours, were able to maintain consistent care despite staff shortages, unlike smaller outpatient clinics, which faced stricter scheduling constraints. Our results reaffirmed the robustness of our system in providing high-quality care even during exceptional circumstances. A key procedural change which has been adopted is a tiered classification system to prioritize surgeries based on urgency, which continues to guide our scheduling practices.

However, it is important to acknowledge the limitations of the study. The analysis was retrospective and based on data from a single tertiary center, which may not fully represent the broader eye care providers. Additionally, the sample size was relatively small, and further research with larger cohorts and multi-center studies is needed to validate these findings and provide a more comprehensive understanding of the impact of the COVID-19 pandemic on the diagnosis and management of FTMH as well as on other ocular diseases in times of a national lockdown due to a pandemic. In our retrospective study, cataract surgery was not performed in all phakic eyes during macular hole surgery as some patients opted against receiving an intraocular lens at the time of surgery. Therefore, the lens status was included as a confounding variable in our statistical analysis. It is well-established that PPV accelerates the progression of cataract formation and following cataract surgery is a risk factor for FTMH reopening^32,33^. Recent evidence suggests that both combined and sequential approaches yield comparable functional and morphological outcomes. However, combined surgery did show faster visual recovery compared to the two-stage approach^34,35^.

The strength of our study is the approach to this previously insufficiently researched topic and the standardized examination, analysis and treatment protocol in four specific time intervals. It highlights symptom duration and macular hole size as key factors for visual function and demonstrates that the German healthcare system maintained high-quality in ophthalmological care in large clinics even during lockdown periods admits the COVID-19 pandemic.

## Conclusion

In conclusion, this retrospective analysis suggests that the management of patients with FTMH during the lockdown periods amidst the COVID-19 pandemic was comparable to the control group of the previous year. There were no significant differences in patient characteristics, preoperative BCVA, symptom duration, time from presentation to surgical treatment, surgical techniques, or FTMH dimensions between the two groups. These findings indicate that the COVID-19 pandemic did not significantly influence the diagnosis and treatment of FTMH in a large tertiary center. The only difference that emerged between the two groups was a significantly higher emergency presentation without prior contact to a community-based ophthalmologist during the lockdown periods. However, further research with larger sample sizes addressing various ophthalmological conditions is necessary to validate these results and gain a more comprehensive understanding of the impact of pandemics on the management of ocular diseases.

## Data Availability

The datasets used and/or analysed during the current study are available form the corresponding author on reasonable request.
